# Precancerous Gastric Lesions with *Helicobacter pylori vacA*^+^/*babA*2^+^/*oipA*^+^ Genotype Increase the Risk of Gastric Cancer

**DOI:** 10.1155/2020/7243029

**Published:** 2020-02-18

**Authors:** Theeraya Simawaranon Bartpho, Wareeporn Wattanawongdon, Taweesak Tongtawee, Chatchanok Paoin, Kokiet Kangwantas, Chavaboon Dechsukhum

**Affiliations:** ^1^Translational Medicine Programs, Institute of Medicine, Suranaree University of Technology, Nakhon Ratchasima 30000, Thailand; ^2^School of Surgery, Institute of Medicine, Suranaree University of Technology, Nakhon Ratchasima 30000, Thailand; ^3^School of Oncology, Institute of Medicine, Suranaree University of Technology, Nakhon Ratchasima 30000, Thailand; ^4^School of Pathology, Institute of Medicine, Suranaree University of Technology, Nakhon Ratchasima 30000, Thailand

## Abstract

**Objective:**

The clinical outcomes of gastric diseases such as chronic gastritis, peptic ulcer, and gastric cancer have been attributed to the interplay of virulence factors of *Helicobacter pylori* (*H. pylori*), host genetic susceptibility, and host immune responses. This study investigated the presence of *cagA*, *vacA*, *iceA*2, *babA*2, and *oipA* genes and their association with clinical outcomes.

**Methods:**

Chronic gastritis, atrophic gastritis, and intestinal metaplasia specimens were obtained from patients who underwent endoscopy and surgical resection between January 2017 and December 2018; specimens from gastric cancer patients treated between January 2014 and December 2018 were also added. *H. pylori*), host genetic susceptibility, and host immune responses. This study investigated the presence of *cagA*, *vacA*, *iceA*2, *babA*2, and *oipA* genes and their association with clinical outcomes. *H. pylori*), host genetic susceptibility, and host immune responses. This study investigated the presence of

**Results:**

*H. pylori*), host genetic susceptibility, and host immune responses. This study investigated the presence of *vacA*, *babA*2, and *oipA* genes and their association with clinical outcomes. *vacA*, *babA*2, and *oipA* genes and their association with clinical outcomes. *P*=0.033, OR = 2.64; 95% CI = 1.44–4.82, *P*=0.033, OR = 2.64; 95% CI = 1.44–4.82, *P*=0.033, OR = 2.64; 95% CI = 1.44–4.82, *H. pylori vacA*^+^/*babA*2, and *oipA* genes and their association with clinical outcomes. *P*=0.033, OR = 2.64; 95% CI = 1.44–4.82,

**Conclusion:**

In this present study, we reported on the virulence genes of *H. pylori* infection to reveal their association with increased risk of chronic gastritis, precancerous gastric lesions, and gastric cancer. Precancerous gastric lesions with *H. pylori vacA*+/*babA*2+/*oipA*+ genotype increased the risk of gastric cancer.*H. pylori*), host genetic susceptibility, and host immune responses. This study investigated the presence of *H. pylori vacA*^+^/*babA*2, and *oipA* genes and their association with clinical outcomes.

## 1. Introduction

*Helicobacter pylori* (*H. pylori*) is a spiral-shaped Gram-negative bacterium that selectively colonizes the gastric mucosa of the humans in more than half of the world's population [1]. *H. pylori* infection has been proven to be highly associated with the development of a variety of gastric diseases such as chronic gastritis, peptic ulcer disease (PUD), mucosal associated lymphoid tissue (MALT), and gastric cancer (GC) [2–4]. These different clinical outcomes have been attributed to the interplay of several factors, including virulence factors of *H. pylori*, host genetic susceptibility, and host immune responses to *H. pylori* infection [5–7]. Several virulence factors have been proposed for *H. pylori* infections; they include adapting to different tissues like urease and flagella, using adhesins such as blood-antigen binding protein A (BabA) and outer inflammatory protein A (OipA), and toxins that damage host tissues such as cytotoxin-associated gene A (CagA) and vacuolating cytotoxin (VacA). Another putative virulence factor is that induced by contact with epithelium A (IceA) [8, 9].

The BabA is a protein for Lewis b binding activity on human gastric epithelial cells. Three *bab* alleles; *babA*1, *babA*2, and *babB* genes have been identified, but only the *babA*2 gene product is functional [10, 11]. BabA2 is associated with an increased risk of PUD and GC [12–14]. The OipA is one of the porin proteins and proinflammatory proteins associated with severe neutrophil infiltration in IL-8 induction and gastric colonization [15]. It contributes to the pathogenesis and is associated with the elevated risks of PUD and GC [16–18]. The *cagA* gene is located at the end of the cag pathogenicity island (cagPAI), encodes a type IV secretion system (T4SS) that is functional for translocating bacterial effectors into host cytoplasm, and triggers the manipulation of cell signaling pathways and also the induction of the proinflammatory cytokines, specifically interleukin IL-8 [19, 20]. It is associated with severe clinical diseases in PUD and GC [21–24]. The VacA is a pore-forming toxin, which causes progressive vacuolation and injury to the gastric epithelium [25]. It is associated with an increased risk of PUD and GC [26, 27]. Specific allelic types in the *vacA* gene are signal (*s*1, *s*2) and the middle regions (*m*1, *m*2) due to sequence heterogeneity [28]. The variation in cytotoxic activities in relation to *H. pylori*-related diseases and gastric mucosal changes is considered the cause of different strains [29]. The *iceA* gene is induced by contact with epithelium and has been considered as a marker for PUD [30]. It has two main allelic variants, *iceA*1 and *iceA*2 [31]. It has been found that *iceA*1 is the predominant subtype in East Asia, while *iceA*2 is the predominant subtype in the USA and Columbia [32]. Although several studies have reported different results, the *iceA*2 gene was detected to be the predominant genotype [33, 34].

In intestinal types of GC, *H. pylori* infection triggers a multistep progression from chronic gastritis, atrophic gastritis (AG), intestinal metaplasia (IM), and finally to GC [35]. Several studies suggest that AG and IM are the major precursor gastric lesions of intestinal-type GC and elevate the risk of GC [36–38]. Moreover, AG and IM increased the risk of intestinal-type GC exponentially when compared with other risk factors [39]. From this background, *H. pylori* infection is thought to be involved in chronic gastritis, precancerous gastric lesions, and GC; however, the relationship between virulence status and its association with these clinical outcomes has not been well reported and are not fully understood in Asian countries. Thus, the aim of this study was to investigate the *H. pylori* virulence genes including *cagA*, *vacA*, *iceA*2, *babA*2, and *oipA* of patients with chronic gastritis, precancerous gastric lesions, and GC, and to determine whether the virulence genes are associated with the risk of chronic gastritis, precancerous gastric lesions, and GC.

## 2. Material and Methods

### 2.1. Patients and Specimens

Patients were subjected to esophagogastroduodenoscopy (EGD) (Olympus Corp., Tokyo, Japan), which was carried out using an upper GI video endoscope (Olympus EVIS EXERA III, CV-190). Gastric tissue biopsies of chronic gastritis, AG, and IM were obtained between January 2017 and December 2018, and GC biopsies were obtained between January 2014 and December 2018. Surgical resection was performed at the Suranaree University of Technology Hospital, Buriram Hospital Medical center, or Surin Hospital Medical center in the Northeastern region of Thailand. Written informed consent was obtained from all patients, and the study protocol was approved by the Ethics Committee for Research Involving Human Subjects, Suranaree University of Technology (EC-59-45 and EC 16-2560). The whole stomach was examined and biopsies were conducted using the site-specific biopsy technique [40]. All biopsies were directly tested for *H. pylori* infection by using the rapid urease test (RUT) kit (Pentland Medical, Edinburgh, UK). The methods were carried out in accordance with good clinical practice and the guidelines of the Declaration of Helsinki [41]. Histological determinations were subsequently examined by a pathologist. The patient retrospective cohort included 70 cases, which were used to analyze the association between overall survival (OS) and genotype combinations.

### 2.2. DNA Extraction

DNA extraction from fresh tissues of chronic gastritis, AG, and IM was performed using the QIAamp DNA mini kit (Qiagen, Düsseldorf, Germany), and the tissues of GC were formalin-fixed and paraffin-embedded (FFPE) using xylene and hydrate in 100% ethanol and subsequently by using QIAamp DNA FFPE tissue kit (Qiagen, Düsseldorf, Germany) according to the manufacturer's instructions. Genomic DNA was purified from the tissue lysate using the QIAamp spin column and eluted. The DNA concentration and purity were determined using a DS-11+ spectrophotometer (Denovix, Wilmington, Delaware, USA) and stored at −20°C.

### 2.3. Real-Time Polymerase Chain Reaction (Real-Time PCR)

*H. pylori* infection 16*s rRNA* and *ureA* genes were identified. *H. pylori*-positive samples were used to determine the virulence genes (*cagA*, *vacA*, *iceA*2, *babA*2, and *oipA*) using real-time PCR. The primers for 16*s rRNA*, *ureA*, *cagA*, *vacA*, *iceA*2, *babA*2, and *oipA* (Integrated DNA Technologies, Coralville, IA, USA) are shown in Table 1. Briefly, DNA samples were used as templates in the amplification reactions. The real-time PCR was performed according to the manufacturer's protocol in a final volume of 20 *μ*L containing DNA template, 2X SYBR Green PCR Master Mix (Roche Applied Science, Mannheim, Germany), and 50 pmol of each primer using a Light Cycler® 480 Instrument (Roche diagnostics, Neuilly sur Seine, France). The PCR conditions used in this study were as follows: preincubation at 95°C for 5 min, 45 cycles of amplification (10 s of denaturation at 95°C, 10 s of annealing at Tm of specific primer, and 10 s of extension at 72°C). Each sample was performed in duplicates reactions for standard. All data were analyzed using the Light Cycler 480 software, version 1.5 (Roche diagnostics, Neuilly sur Seine, France).

### 2.4. Statistical Analysis

The differences between the virulence genes of *H. pylori* infection for the patient's demographic data were determined using ANOVA. The associations between virulence genes and clinical outcomes and risks of GC were evaluated using the univariate regression model analysis. Odds ratios (OR) and 95% confidence intervals (CI) were calculated using the multivariate regression model analysis. Survival analysis was performed using the Kaplan–Meier method and the overall survival differences were analyzed using the log-rank test. A *P* value of less than 0.05 was considered statistically significant. All statistical analyses were carried out using SPSS for Windows (version 20.0; IBM Corp., Armonk, NY, USA).

## 3. Results

### 3.1. Detection of *H. pylori cagA*, *vacA*, *iceA*2, *babA*2, and *oipA* Virulence Genes in Chronic Gastritis, Precancerous Gastric Lesions, and Gastric Cancer

**Figure 1:**
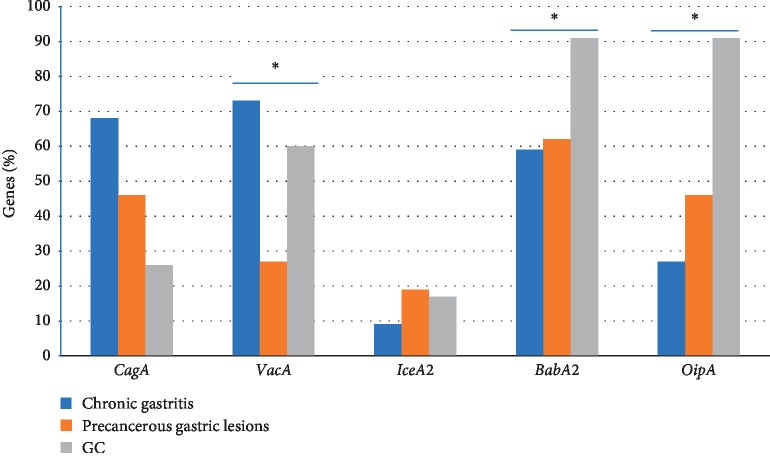
The frequency of cagA, vacA, iceA2, babA2, and oipA genes in each clinical outcome.

A total of 200 *H. pylori*-positive samples was examined for 16*s rRNA* and *ureA* gene detection; out of which 166 patients (83%) were positive for both 16*s rRNA* and *ureA* genes. These patients were divided into three groups: chronic gastritis (*n* = 44), precancerous gastric lesions including AG and IM (*n* = 52), and GC (*n* = 70). All patients were not significantly different in age and gender. The patient's demographic data are summarized in Table 2. The *vacA* (73%) gene was mainly present in chronic gastritis followed by *cagA* (68%), *babA*2 (59%), *oipA* (27%), and *iceA*2 (9%). The *babA*2 (62%) gene was mainly present in precancerous gastric lesions followed by *cagA* and *oipA* (46%), *vacA* (27%), and *iceA*2 (19%). Interestingly, *babA*2 and *oipA* (91%) were almost present in GC followed by *vacA* (60%), *cagA* (26%), and *iceA*2 (16%). The *iceA*2 gene was the lowest frequency of detection in all clinical outcomes. The frequency of *cagA*, *vacA*, *iceA*2, *babA*2, and *oipA* genes is shown in Figure 1. The presence of *vacA*, *babA*2, and *oipA* genes was significantly different between clinical outcomes (*P*=0.036, 0.042 and 0.039, respectively).

### 3.2. Association between the Presence of *vacA*, *babA*2, and *oipA* Genes and Clinical Outcomes

The association between the presence of *vacA*, *babA*2, and *oipA* genes and clinical outcomes was assessed. Among chronic gastritis, *vacA* was present in *H. pylori* infection and was associated with significantly increased risk of chronic gastritis (OR = 2.14, 95% CI = 1.62–4.46, *P*=0.036). The *vacA*, *babA*2, and *oipA* genes were present in patients, but they were not associated with increased risk of precancerous gastric lesions. Additionally, *vacA*, *babA*2, and *oipA* genes were associated with increased risk of GC (OR = 1.23; 95% CI = 1.13–3.32; *P*=0.033, OR = 2.64; 95% CI = 1.44–4.82, *P*=0.024 and OR = 2.79; 95% CI = 1.58–5.41; *P*=0.031, respectively) (Table 3).

### 3.3. *H. pylori vacA*^+^/*babA*2^+^/*oipA*^+^ Genotype Conferred Increased Risk of GC

We examined the virulence combinations based on the analysis of *vacA*, *babA*2, and *oipA* genotypes. Precancerous gastric lesions infected with *vacA*^+^/*babA*2^+^, *vacA*^+^/*oipA*^+^, *babA*2^+^/*oipA*^+^, and *vacA*^+^/*bibA*2^+^/*oipA*^+^ genotypes were 3.85%, 3.85%, 26.92%, and 11.54%, whereas GC were 2.86%, 2.86%, 34.29%, and 51.43%, respectively (Table 4). Interestingly, precancerous gastric lesions infected with *H. pylori* genotype combination of *vacA*^+^/*bibA*2^+^/*oipA*^+^ were highly significantly associated with increased risk of GC (OR = 3.85, 95% CI = 1.67–5.77, *P*=0.021), but not *vacA*^+^/*babA*2^+^, *vacA*^+^/*oipA*^+^, and *bibA*2^+^/*oipA*^+^ genotypes. Chronic gastritis was not associated with the development of precancerous gastric lesions or GC when infected with any *H. pylori* genotype combination (data not shown).

### 3.4. Overall Survival of GC Patients with *H. pylori* Genotype Combination Infections

**Figure 2:**
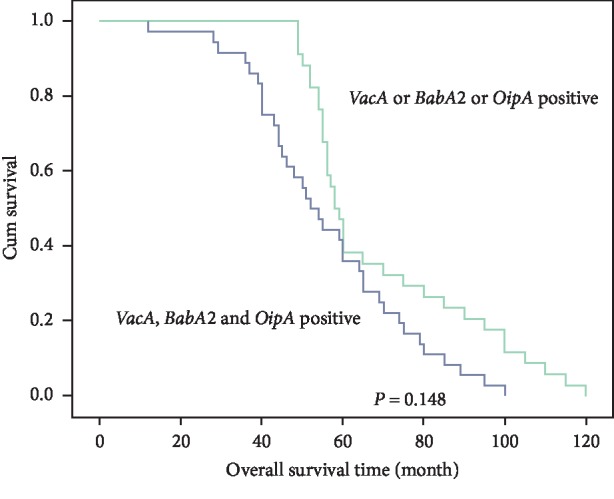
Overall survival time of gastric cancer patients infected with *H. pylori* two-genotypes combination (*vacA*^+^/*babA*2^+^, *vacA*^+^/*oipA*^+^ and *babA*2^+^/*oipA*^+^) and *H. pylori* three-genotypes combination (*vacA*^+^/*babA*2^+^/*oipA*^+^).

We examined the overall survival of GC patients that were infected with *H. pylori* two-genotype strains (*vacA*^+^/*babA*2^+^, *vacA*^+^/*oipA*^+^, and *babA*2^+^/*oipA*^+^) and *H. pylori* three-genotype strain (*vacA*^+^/*babA*2^+^/*oipA*^+^). The mean survival time for patients with *H. pylori* two-genotype infection was 69.52 ± 3.72 months and with *H. pylori* three-genotype infection was 56.16 ± 3.23 months. However, the overall survival of patients infected with *vacA*^+^/*babA*2^+^/*oipA*^+^ genotype strain was decreased, but there was no statistically significant difference between the two groups (*P*=0.148; Figure 2).

## 4. Discussion

Our study was a cross-sectional study, which investigated the presence of *cagA*, *vacA*, *iceA*2, *babA*2, and *oipA* genes in *H. pylori* infected patients with chronic gastritis, precancerous gastric lesions, and GC. The rate of *H. pylori* infection in this study was 83%, which was comparable with a retrospective study in the Northeastern region of Thailand [49] and a prospective study in Japan [36]. This study was the first report on the associations between virulence genes and the risk of chronic gastritis, precancerous gastric lesions, and GC in Thailand. Our results revealed that the *vacA* gene was associated with chronic gastritis whereas *vacA*, *babA*2, and *oipA* genes were associated with increased risk of GC. These indicated that *vacA* and *babA*2 genes influenced chronic gastritis and precancerous gastric lesions, respectively. Meanwhile, the *babA*2 and *oipA* genes had virulence potential on GC development. The *babA*2 and *oipA* genes were present mostly in *H. pylori*-positive GC; it seems *vacA*, *babA*2, and *oipA* genes exhibited different levels of virulence. It is probable that vacA alone was not directly associated with gastric carcinogenesis. Although, vacA effects on disruption of gastric epithelial barrier function and modulation of the inflammatory response, vacA also suppresses the activation of ERK1/2 mitogen-activated protein (MAP) kinase suggesting that *H. pylori* can avoid the induction of excess cellular damage and maintain long-term colonization [5]. Therefore, vacA individual may develop chronic gastritis.

*H. pylori* infection induced cell-mediated immunity. Th1 cells play a central role in *H. pylori* immune response. Predominant Th17 expression was positively correlated with the degree of immunopathologic reactions resulting in peptic ulcers [6]. Furthermore, increasing of T-bet + cells and the mucosal INF-*γ* expression related to the degree of *H. pylori* density in infected patients can lead to ulcer or GC [7]. In addition, the roles of babA2 and oipA proteins might have the potential to exert pressure on *H. pylori* by enhancing the production of free radicals that cause mutations in target cells and the neoplastic clones are established [39]. Tumor necrosis factor alpha (TNF-*α*) plays major roles in the growth, invasion, and metastasis of neoplasm called a perigenetic pathway [50]. The TNF-*α* inducing protein (Tip*α*) from *H. pylori* binds to and enters the nucleus through a specific binding molecule, which acts as a carcinogen [51] and contributes to GC. Although, cagA is recognized as an oncoprotein and confers oncogenesis, the presence of *cagA* was not associated with any clinical outcome, suggesting that the *cagA* gene present in East Asian strains might not be influential of its risk enough.

Interestingly, genotype combination was associated with GC. The precancerous gastric lesions with *H. pylori vacA*^+^/*babA*2^+^/*oipA*^+^ genotype infection had high association with 4.3-fold increased risk of GC development. The *babA*2 gene has been strongly associated with *vacA* and increased risk of GC development [24]; however, in this study, the *babA*2^+^/*vacA*^+^ genotype did not have increased risk of GC. Taken together, oipA was associated with higher neutrophil activity and IL-8 secretion and showed toxic effects by an apoptosis-triggered cascade via signaling that affected the Bax/Bcl-2 protein ratio and cleaved-caspase 3 level, leading to a mitochondrial apoptotic cascade [52–54]. These findings suggest that the *vacA*^+^/*babA*2^+^/*oipA*^+^genotype may contribute to the genotoxicity caused by DNA damage and aberrant methylation of genes in *H. pylori*-related gastric carcinogenesis. A cohort study with long-term follow-up demonstrated that infection with *cagA* genotype was associated with increased risk of precancerous gastric lesions progression [55, 56]. Therefore, *vacA*, *babA*2, and *oipA* have been implicated in the development of GC. Regarding precancerous gastric lesions after *H. pylori* eradication, *H. pylori*-induced chronic inflammation can provide the seed of cascade leading to GC, which can continuously progress even in the absence of *H. pylori* [57]. The patients with *H. pylori* and IM have more than 6.4-fold increased risk of GC than that of the patients with *H. pylori* but without IM [36]. However, 2.9% GC was developed in individuals during the mean follow-up of 7.8 years [36]. Therefore, precancerous gastric lesions patients infected with the *H. pylori vacA*^+^/*babA*2^+^/*oipA*^+^ genotype were prone to developing GC compared with patients infected with other combination genotypes. However, in the present study, the overall survival time of GC patients with the *H. pylori vacA*^+^/*babA*2^+^/*oipA*^+^ genotype infection was not reduced.

The limitations of the present study were that subanalysis for the precancerous gastric lesions was not performed and the number of patients involved was small and there was no regular follow-up on them. Furthermore, several virulence factors of *H. pylori* were not investigated. The expression of vacA, babA2, and oipA protein should be evaluated in a future study of GC carcinogenesis to determine the underlying mechanisms associated with GC development in precancerous gastric lesions patients.

## 5. Conclusion

This study provided important information regarding the presence of virulence genes in different clinical outcomes of *H. pylori* infection. Precancerous gastric lesions of patients infected with *H. pylori vacA*^+^/*babA*2^+^/*oipA*^+^ genotype infection have an increased risk of GC. The *H. pylori vacA*^+^/*babA*2^+^/*oipA*^+^ genotype might prove helpful in predicting individuals in the high-risk group of GC in the Thai population.

## Figures and Tables

**Table 1:** Primers used for the amplification of *H. pylori* genes.

Primer	Forward	Reverse	Reference
16*s rRNA*	GGAGTACGGTCGCAAGATTAAA	CTAGCGGATTCTCTCAATGTCAA	[42]
*UreA*	CGTGGCAAGCATGATCCAT	GGGTATGCACGGTTACGAGTTT	[43]
*CagA*	GAGTCATAATGGCATAGAACCTGAA	TTGTGCAAGAAATTCCATGAAA	[44]
*VacA*	CTCCAGAAGGCACACCAATAA	TGGCTTCCACTTCCCCATTAA	[45]
*IceA*2	GTTGTCGTTGTTTTAATGAA	GTCTTAAACCCCACGATTAAA	[46]
*BabA*2	CCAAACGAAACAAAAAGCGT	GCTTGTGTAAAAGCCGTCGT	[47]
*OipA*	GTTTTTGATGCATGGGATTT	GTGCATCTCTTATGGCTTT	[48]

**Table 2:** Demographic characteristics.

	Chronic	Precancerous lesions	Gastric cancer	*P* value
	(*n* = 44)	(*n* = 52)	(*n* = 70)	
Age: mean	43 ± 1.6	46 ± 2.4	52 ± 1.2	0.192
Gender: male/female (%)	63.6/32.4	46.2/43.8	45.2/44.8	0.082
Pathological characteristic of gastric cancer patients (*n* = 70)				
Location of tumor *n* (%)				
Upper				12 (17.15)
Middle				36 (51.42)
Lower				22 (31.43)
Tumor size *n* (%)				
<70 mm				18 (25.72)
≥70 mm				52 (74.28)
Histologic type *n* (%)				
Differentiated				54 (77.14)
Undifferentiated				16 (22.86)
Lymphatic invasion *n* (%)				
Absent				46 (65.71)
Present				24 (34.29)
Vascular invasion *n* (%)				
Absent				62 (88.57)
Present				8 (11.43)
Pathological T stage *n* (%)				
T1-T2				22 (31.43)
T3-T4				48 (68.57)
Pathological TNM stage *n* (%)				
I				8 (11.43)
II				14 (20.0)
III				36 (51.43)
IV				12 (17.14)
Residual tumor *n* (%)				
No residual tumor				52 (74.28)
Microscopic				6 (8.57)
Gross (unresectable)				12 (17.14)
CEA *n* (%)				
<5.0 (ng/ml)				42 (60.0)
≥5.0 (ng/ml)				28 (34.29)

Comparisons between the groups were done by using ANOVA. *P* < 0.05 considered as statistically significant.

**Table 3:** Virulence gene in association with clinical outcomes.

Gastric mucosa pathology/Virulence gene	Chronic	Precancerous	OR; 95% CI	*P* value	Precancerous	GC	OR (95% CI)	*P* value
	(*n* = 44)	(*n* = 52)			(*n* = 52)	(*n* = 70)		
*VacA*	32 (73%)	14 (27%)	2.14 (1.62–4.46)	0.036	14 (27%)	42 (60%)	1.23 (1.13–3.32)	0.033
*BabA*2	26 (59%)	32 (62%)	0.77 (0.56–0.94)	0.833	32 (62%)	64 (91%)	2.64 (1.44–4.82)	0.024
*OipA*	12 (27%)	24 (46%)	0.69 (0.49–0.82)	0.546	24 (46%)	64 (91%)	2.79 (1.58–5.41)	0.031

Multivariate regression model analysis used to analyze the data. OR: odds ratio; CI: confidence interval. Significance is set at *P* < 0.05.

**Table 4:** Virulence genotype combination in association with clinical outcomes.

Virulence gene			Precancerous gastric lesion (%)	GC (%)	OR (CI 95%)	*P* value
*VacA*	*BabA*2	*OipA*				
**+**	+	−	2 (3.85)	2 (2.86)	0.72 (0.42–0.97)	0.634
**+**	−	+	2 (3.85)	2 (2.86)	0.72 (0.42–0.97)	0.634
−	+	+	14 (26.92)	24 (34.29)	0.7 (0.37–0.96)	0.091
**+**	+	+	6 (11.54)	36 (51.43)	4.28 (1.82–7.41)	0.021

Multivariate regression model analysis used to analyze the data. OR: odds ratio; CI: confidence interval. Significance is set at *P* < 0.05.

## Data Availability

No data were used to support this study.
